# Lysine acetylation regulates the AT-rich DNA possession ability of H-NS

**DOI:** 10.1093/nar/gkad1172

**Published:** 2023-12-07

**Authors:** Yabo Liu, Mengqing Zhou, Yifan Bu, Liang Qin, Yuanxing Zhang, Shuai Shao, Qiyao Wang

**Affiliations:** State Key Laboratory of Bioreactor Engineering, East China University of Science and Technology, Shanghai 200237, China; State Key Laboratory of Bioreactor Engineering, East China University of Science and Technology, Shanghai 200237, China; State Key Laboratory of Bioreactor Engineering, East China University of Science and Technology, Shanghai 200237, China; New Product R&D, GenScript Biotech Corporation, Nanjing 211100, China; Laboratory for Marine Fisheries Science and Food Production Processes, Qingdao National Laboratory for Marine Science and Technology, Qingdao, China; Shanghai Engineering Research Center of Maricultured Animal Vaccines, Shanghai 200237, China; Laboratory of Aquatic Animal Diseases of MOA, Shanghai 200237, China; State Key Laboratory of Bioreactor Engineering, East China University of Science and Technology, Shanghai 200237, China; Shanghai Engineering Research Center of Maricultured Animal Vaccines, Shanghai 200237, China; Laboratory of Aquatic Animal Diseases of MOA, Shanghai 200237, China; State Key Laboratory of Bioreactor Engineering, East China University of Science and Technology, Shanghai 200237, China; Laboratory for Marine Fisheries Science and Food Production Processes, Qingdao National Laboratory for Marine Science and Technology, Qingdao, China; Shanghai Engineering Research Center of Maricultured Animal Vaccines, Shanghai 200237, China; Laboratory of Aquatic Animal Diseases of MOA, Shanghai 200237, China

## Abstract

H-NS, the histone-like nucleoid-structuring protein in bacteria, regulates the stability of the bacterial genome by inhibiting the transcription of horizontally transferred genes, such as the type III and type VI secretion systems (T3/T6SS). While eukaryotic histone posttranslational modifications (PTMs) have been extensively studied, little is known about prokaryotic H-NS PTMs. Here, we report that the acetylation of H-NS attenuates its ability to silence horizontally transferred genes in response to amino acid nutrition and immune metabolites. Moreover, LC−MS/MS profiling showed that the acetyllysine sites of H-NS and K120 are indispensable for its DNA-binding ability. Acetylation of K120 leads to a low binding affinity for DNA and enhances T3/T6SS expression. Furthermore, acetylation of K120 impairs the AT-rich DNA recognition ability of H-NS. In addition, lysine acetylation in H-NS modulates *in vivo* bacterial virulence. These findings reveal the mechanism underlying H-NS PTMs and propose a novel mechanism by which bacteria counteract the xenogeneic silencing of H-NS.

## Graphical Abstract

**Graphical Abstract:**
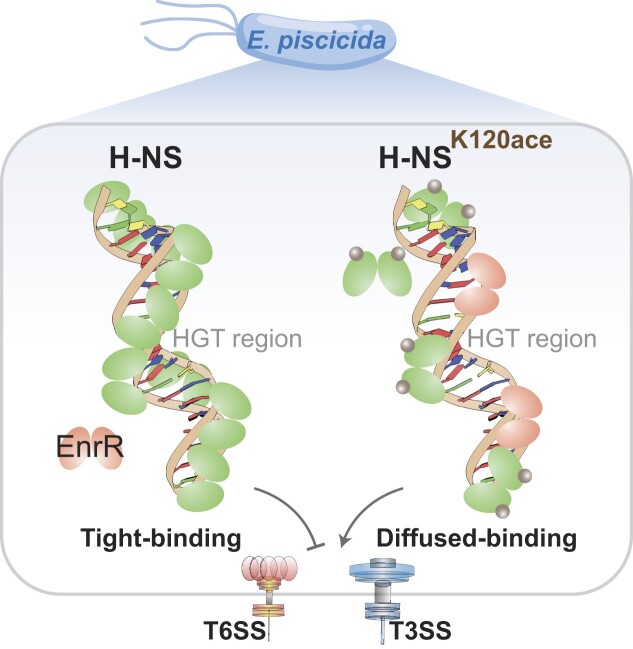

## Introduction

Horizontal gene transfer (HGT) is a tremendous driving force for bacterial evolution, facilitating the acquisition of antibiotic resistance genes, two-component systems, secretory systems and other genetic elements to improve environmental adaptation (1). Given that excessive HGT can cause genomic disruption, bacteria employ nucleoid-associated proteins such as H-NS to maintain genome stability and silence horizontally transferred genes (2). H-NS, a highly expressed protein with approximately 20 000 copies per cell, primarily binds to AT-rich DNA in the narrow minor groove and causes topological changes, thus obstructing the transcription process of RNA polymerase (RNAP) (3–5).

The silencing of heterologous genes by H-NS consistently prevents the utilization of the advantages of HGT. Therefore, bacteria have developed countersilencing proteins such as PhoP (6), LuxR (7), SsrB (8) and EnrR (9) to expel H-NS (10). We previously reported that EnrR’s N-terminal extension mediates minor groove contacts and cooperative DNA binding in the distal DNA duplex to form a DNA loop, leading to compaction for occlusion of H-NS binding to the DNA minor grooves (9). In addition to countersilencing proteins, H-NS itself undergoes a variety of changes to alleviate xenogeneic silencing in response to environmental cues. Osmotic pressure can change the conformation of H-NS (11), and temperature shifts modulate the phosphorylation of H-NS in the N-terminal oligomeric domain, affecting its DNA binding function (12,13).

Acetyllysine in bacteria has been implicated in various biological processes, including carbon source utilization (14,15), ammonium assimilation (16), virulence (17) and CRISPR−Cas activity (18). Furthermore, lysine acetylation at different sites within bacterial proteins has different regulatory functions. In PhoP of *Salmonella enterica* serovar Typhimurium, acetylation at K99 affects its dimerization (19), acetylation at K102 influences its phosphorylation (20) and acetylation at K201 impacts its DNA binding ability (17). H-NS is a polylysine protein (total amino acids ∼135, lysines ∼11) that undergoes various PTMs, such as acetylation, deamidation, methylation, phosphorylation and succinylation (21). A large number of H-NS acetyllysine sites identified by mass spectrometry have attracted increasing attention; however, the vital role of H-NS acetylation has yet to be experimentally confirmed (21–24).

*Edwardsiella piscicida*, a gram-negative pathogen, constantly shuttles between water and aquatic animals, which facilitates extensive HGT within the genome of *E. piscicida* (25). *E. piscicida* EIB202 has 24 genomic islands (GIs) acquired through HGT (26), including the type III secretion system (T3SS) of GI7 and the type VI secretion system (T6SS) of GI17, which are crucial to ensuring bacterial intracellular survival (27,28). The T3SS of *E. piscicida* shares homology with the T3SS of *S*. Typhimurium SPI-2 and is regulated by the SsrA/B homologous two-component system proteins EsrA/B (28). In *E. piscicida*, H-NS acts as an inhibitor of both the T3SS and T6SS and is expelled by the countersilencing protein EnrR during infection (9). In addition, acetylomics analysis of *E. piscicida* identified 1511 lysine acetylation sites on 589 proteins, which are predominantly involved in the tricarboxylic acid cycle, pyruvate metabolism and antimicrobial resistance pathways (29). Here, we characterized H-NS as a lysine acetylation protein and identified its acetyllysine sites under favorable amino acid nutrition conditions. Specifically, acetylation affects the DNA-binding ability of H-NS and its possession of an AT-rich DNA region. The impaired DNA-binding ability makes H-NS more susceptible to being expelled by countersilencing proteins, thereby facilitating the activation of T3/T6SS. Collectively, these findings show that acetylation may serve as an alternative countersilencing mechanism for H-NS.

## Materials and methods

### Bacterial and cell strains, media and culture conditions

The bacterial strains, plasmids and primers used in this study are listed in Supplementary Table S1. Normally, *E. coli*, *E. piscicida* and *S*. Typhimurium strains were cultured in lysogeny broth (LB) broth at 37 or 30°C. To induce T3/T6SS production, *E. piscicida* was grown statically at 30°C in DMEM. When needed, ampicillin (Amp, 50 μg/ml), colistin (Col, 12.5 μg/ml), chloramphenicol (Cm, 34 μg/ml), gentamicin (Gm, 50 μg/ml), streptomycin (Str, 50 μg/ml) and kanamycin (Kan, 50 μg/ml) were added.

### Genetic engineering of bacteria

Genetic engineering (in-frame deletion, point mutation, fusion tag) was achieved through *sacB*-based allelic exchange as previously described (28). Briefly, upstream and downstream fragments were amplified by polymerase chain reaction (PCR) followed by ligation into the suicide vector pDMK. The resulting plasmid was transformed into SM10 λ*pir* and subsequently introduced into *E. piscicida* and *S*. Typhimurium via conjugation. The insertion mutants with single crossover recombination events were selected on LB plates containing Cm, Kan, and Str. Double-crossover mutants were selected on LB plates containing 10% sucrose. Complementation and overexpression plasmids were constructed with pUT plasmids, which were introduced into *E. piscicida* by electroporation at 2.5 kV for 2.5 ms.

### Quantitative RT-PCR (qRT−PCR)

*E. piscicida* wild type (WT) and mutants were statically cultured at 30°C for 14 h. RNA samples were extracted with an RNA isolation kit as previously described (30). In total, 1 μg of each RNA sample was utilized with One-Step gDNA Removal and cDNA Synthesis SuperMix (Transgene, China). Three independent qRT−PCR experiments were performed using specific primer pairs (Supplementary Table S1). The comparative ΔΔ*C_T_* method was employed to determine the relative quantities of each transcript, and the housekeeping gene *dnaA* was used as an internal control.

### Extracellular protein (ECP) assays and western blot analysis

*E. piscicida* WT and mutants were statically cultured at 30°C for 14 h. The supernatants from identical amounts of bacteria were separated by SDS−PAGE and subsequently silver stained (Yeasen, China). The gel was imaged by a scanner (Epson V500, Japan).

For Western blot analysis, proteins separated by SDS-PAGE from equal amounts of bacteria were transferred onto a PVDF membrane. The membrane was then blocked with 10% nonfat milk in TBST for 2 h at room temperature. Subsequently, the membrane was probed with specific antibodies against His (Yeasen, China), RNA polymerase α subunit (Huabio, China), EseB (Huabio, China), EvpP (Huabio, China) and acetyllysine (Jingjie PTM Biolab, PTM101, China) at 4°C for 14 h. After washing with TBST three times, the membrane was incubated with the corresponding secondary antibodies at room temperature for 1 h. Following three washes with TBST, the signals on the membrane were detected with Immobilon Western Chemiluminescent HRP Substrate (Beyotime, China). The numbers correspond to the normalized protein abundance relative to the leftmost lane or DMEM group. The normalized protein abundance is the ratio of each protein band relative to the loading control, which was quantified with ImageJ.

### Protein purification

The recombinant proteins were prepared as previously described (9). For EMSA, *hns*, *hns*^K120^ variants, and *enrR* were inserted into pETduet-1 mcs1 to form a recombinant plasmid and then transformed into *E. coli* BL21 for protein production. When the OD_600_ of the bacterial culture reached 0.6, expression was induced with 0.2 mM isopropyl-β-d-1-thiogalactopyranoside (IPTG), and the cells were grown at 18°C for 14 h. Following cell lysis using a high-pressure cracker (800–900 bar), cell lysates were collected by centrifugation at 12 000 × g for 30 min at 4°C and loaded onto a Ni-affinity column (Yeasen, China). Contaminant proteins were removed using Buffer A (20 mM Tris, 500 mM NaCl, 20–80 mM imidazole, pH 8.0), and the protein of interest was eluted using Buffer A containing 500 mM imidazole.

For the detection of H-NS acetyllysine in *E. piscicida* and *S*. Typhimurium H-NS, a His_6_-tag sequence was fused to the C-terminus of the chromosomal-encoded H-NS using the *sacB*-based allelic exchange method. During the purification process, no IPTG was added to the growth medium, and 10 mM nicotinamide was added to buffer A.

For the expression of site-specifically acetylated H-NS protein, *E. coli* BL21 was transformed with the plasmids pAcKRS-3 and pCDF-PylT-*hns*^K120^ (TAG) as previously described (17,31). A 500 ml aliquot of LB was inoculated with 5 ml of overnight cultured *E. coli* and incubated at 37°C. When the OD_600_ reached 1.5, the culture was supplemented with 500 ml of fresh LB with 20 mM acetyllysine. Protein expression was induced by adding 0.5 mM IPTG and incubating for 8 h at 25°C.

### LC−MS/MS analysis

The purified H-NS proteins from *E. piscicida* were separated using SDS−PAGE. The stained H-NS bands were cut from the gel and then transferred into 1.5 ml EP tubes. LC−MS/MS analysis was conducted by APTBIO (Shanghai, China). The analysis was performed on a Q Exactive mass spectrometer coupled to an Easy-nLC for 60 min (Thermo Scientific). The mass spectrometer was operated in positive ion mode, and data were analyzed using Mascot 2.2 software (Matrix Science, UK) against the H-NS protein sequence. The parameters (peptide mass tolerance = 20 ppm, MS/MS tolerance = 0.1 Da, enzyme = trypsin, missed cleavage = 2) were used to identify the lysine acetylation site based on a 42 Da increase. The peptides are listed in Supplementary Table S2.

### Electrophoretic mobility shift assay (EMSA)

Briefly, protein samples with a gradient of concentrations were incubated with 20 ng of Cy5-labeled DNA probes in EMSA buffer (150 mM NaCl, 0.1 mM DTT, 0.1 mM EDTA, 200 ng poly(dI:dC) and 20 mM Tris, pH 8.0) at 25°C for 30 min. Subsequently, the mixtures were loaded onto a 10% polyacrylamide gel and subjected to electrophoresis in Tris-glycine buffer at 4°C and 100 V for 80 min. The gels were then imaged using a Typhoon FLA-9500 system (GE Healthcare, USA).

### DNA pull-down bridging assay

The DNA bridging assay was performed as previously described with slight modifications (11). Briefly, streptavidin-coated magnetic beads (30 μl) were resuspended in 60 μl of coupling buffer (20 mM Tris–HCl pH 8.0, 2 mM EDTA, 2 M NaCl, 2 mg/ml BSA, 0.04% Tween 20) containing biotinylated DNA (500 bp) followed by incubation for 20 min at 25°C. The beads were washed three times with incubation buffer (10 mM Tris–HCl pH 8.0, 5% v/v glycerol, 1 mM spermidine). Next, unlabeled 500 bp DNA and H-NS proteins were added to initiate the formation of a bridged DNA−DNA complex, which was incubated for 20 min at 25°C. After incubation, the beads were washed three times and then incubated with 0.1% SDS for 5 min at 95°C. The recovered bridged DNA was then amplified and resolved on an agarose gel. All DNA bridging experiments were performed at least in triplicate.

### Chromatin immunoprecipitation sequence (ChIP-seq) and ChIP-qPCR

Initially, strains chromosomally expressing H-NS-Flag, H-NS^K120Q^-Flag and H-NS^K120R^-Flag were incubated in LB medium or DMEM at 30°C for 14 h. The cultures were treated with 1% formaldehyde at room temperature for 10 min, and the reaction was stopped by adding 125 mM glycine. Next, the bacteria were washed twice with precooled PBS and resuspended in 15 ml of IP buffer (20 mM Tris, 150 mM NaCl, 1 mM EDTA, pH 8.0 and protease inhibitor cocktails). High pressure and ultrasound were used for bacterial cell disruption and DNA fragmentation. The samples were washed with 40 μl of protein G beads and then incubated overnight with 40 μl of Flag beads (Sigma−Aldrich, USA). The beads were sequentially washed twice using low-salt wash buffer, high-salt wash buffer, and TE buffer. The beads were then resuspended in 200 μl of elution buffer and 8 μl of 5 M NaCl, followed by incubation at 65°C for 12 h. The supernatants containing the immunoprecipitated DNA were collected. After treatment with RNase A and proteinase K, ChIP DNA was purified using phenol−chloroform. The sequencing library was constructed using a VAHTS Turbo DNA library prep kit (Vazyme, China) and sequenced on the Nova-Seq platform (Illumina, USA).

After adapter removal and alignment to the genome, the sequencing data were analyzed to identify peaks using MACS2. The AT ratio was calculated based on the call peak result, and fragments were selected for calculation, including 49 bp before and after the peak summit. STREME was used to generate the binding motif (32). The frequency distributions of successive dinucleotides and trinucleotides within each peak were calculated, yielding 4^2^ and 4^3^ possible combinations for dinucleotide and trinucleotide, respectively. The Spearman correlation coefficients between the frequency (dinucleotide and trinucleotide usage deviation) and the corresponding recruitment levels among all peaks were calculated using the Python package SciPy. Positive/negative coefficients (Spearman's rho) are shown in red/blue bars, representing coefficients between the oligonucleotide usage deviations and H-NS recruitment levels in ChIP-seq peaks (9,33). ChIP-seq data were visualized using IGV, TBtools and deepTools software (34,35). The peaks are listed in Supplementary Table S3.

ChIP−qPCR analyses were performed as previously described (30). For each DNA target, the Δ*C*_T_ of the input fraction and IP fraction was calculated and then normalized by dividing it by the corresponding Δ*C*_T_ obtained for the nonspecific *dnaA* intragenic region in the indicated strains. Subsequently, the enrichment ratio was calculated from the ΔΔ*C*_T_ value.

### Cell and zebrafish infection model

Macrophages (J774A.1) were seeded at a density of 2.0 × 10^5^ cells/well in 24-well plates and incubated overnight at 37°C with 5% CO_2_. *E. piscicida* cultures were inoculated into fresh LB medium and statically grown for 14 h at 30°C. Cells were infected with *E. piscicida* at a multiplicity of infection (MOI) of 10, followed by centrifugation at 600 × g for 10 min to facilitate bacterial attachment to cells. After a 2 h infection, the cells were washed twice with PBS. Opti-MEM was added to cell cultures with 50 μg/ml Gm to kill extracellular bacteria, and the cells were further incubated at 35°C with 5% CO_2_ for another 2 h. Subsequently, the cultures were treated with PBS containing 1% Triton X-100 for 30 min to disrupt the cells. Intracellular bacteria were enumerated by serial dilution plating on LB plates.

Overnight cultured strains were harvested by centrifugation at 8000 ×*g* for 2 min and washed twice with PBS. A suspension of 400 CFU of bacteria suspended in 5 μl of PBS was intramuscularly injected into each zebrafish. PBS was used as the negative control. A total of 25 fish were injected with each strain, and fish mortality was monitored four times daily at equal intervals.

### Statistical analyses

Statistical analyses were performed using GraphPad Prism (version 6.0). An unpaired two-tailed Student's *t* test was used for statistical analysis, and differences were considered significant at * *P* < 0.05, ** *P* < 0.01 and *** *P* < 0.001.

## Results

### The essential role of *hns* in survival and T3/T6SS expression

Previously, we established a mariner-based Himar1 transposon library in *E. piscicida* EIB202 and characterized the insertion profile of each gene through high-throughput sequencing (27). Due to the noninsertable events, *hns* is proposed as an essential gene in *E. piscicida* (Supplementary Figure S1A). Similar to the result in *S*. Typhimurium (3), we were unable to obtain a deletion strain of *hns* using *sacB*-based gene editing. To clarify the role of H-NS in *E. piscicid*a, the CRISPR-dCas9 system was used to knock down *hns* expression, and the pUT plasmid was employed to overexpress *hns*. The gRNA-hnsi strain exhibited a significant decrease in *hns* mRNA production compared to the control strain expressing nontarget gRNA, while the overexpression strain exhibited a higher expression level of *hns* (Supplementary Figure S1B). The mRNA expression levels of representative genes of the T3/T6SS, including *esrC* and *eseB* (T3SS genes) and *evpC* and *evpP* (T6SS genes), were measured, and the findings suggested that overexpression of *hns* led to a decrease in T3/T6SS-related gene expression, while knockdown resulted in the upregulation of these genes (Supplementary Figure S1B). Furthermore, Western blot analysis targeting EseB and EvpP in *E. piscicida* confirmed the suppression of H-NS on T3/T6SS expression (Supplementary Figure S1C). The extracellular proteins (ECPs), including EseB/C/D (homologous to *Salmonella* T3SS needle and translocon proteins) and EvpC (a homologous protein of *Pseudomonas aeruginosa* T6SS HCP1), were determined through SDS−PAGE followed by silver staining, further supporting the inhibitory role of H-NS in the regulation of T3/T6SS (Supplementary Figure S1D).

### Acetylation of H-NS in response to amino acid nutrition and immune metabolites

**Figure 1.**
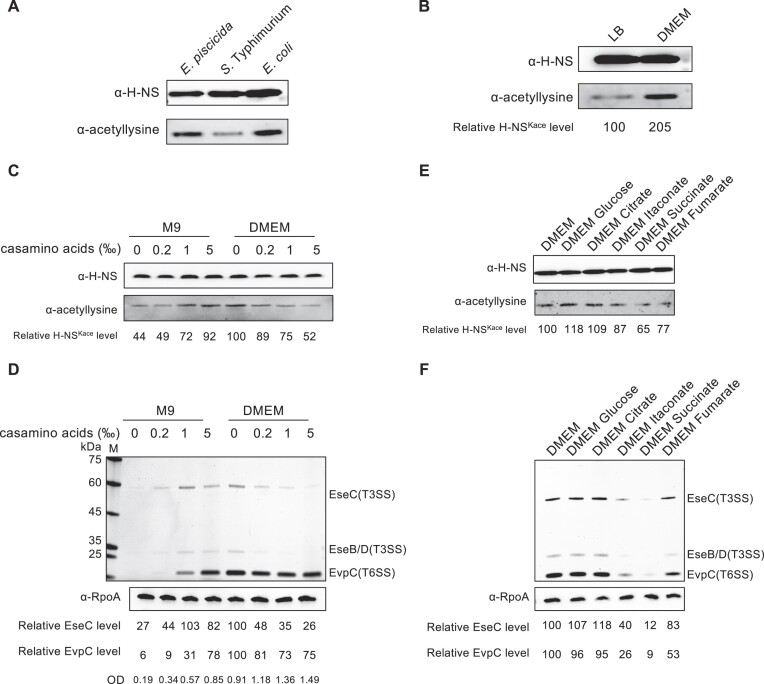
Acetylation of H-NS responds to amino acid nutrition and immune metabolites. (**A**) Acetylation level of H-NS purified from *E. piscicida* (EIB202), *S*. Typhimurium (SL1344) and *E. coli* (DE3). (**B**) Acetylation level of H-NS from *E. piscicida* cultured in LB medium and DMEM. (**C**) Acetylation of H-NS in response to amino acid nutrition. Casamino acids were added in a gradient to increase amino acid nutrition in M9 medium and DMEM. **(E)** Acetylation of H-NS in response to immune metabolites. The indicated metabolites in DMEM were added at a final concentration of 5 mM. The acetylation level of H-NS-His_6_ was determined by the pan anti-acetyllysine antibody, and the anti-His_6_ antibody was used as a loading control. The acetylation levels were determined by Western blotting; the image is representative of at least three independent replicates. The relative acetylation level was calculated as acetylated H-NS divided by total H-NS. The relative acetylation level of H-NS in LB (B) and DMEM (C, E) was set as 100. *** *P* < 0.001 (Student's *t* test). (**D, F**) Extracellular protein profile analysis of T3SS and T6SS expression in (C, E). The supernatants from identical amounts of bacteria were resolved by SDS-PAGE and then silver stained. A blot of RpoA was used as a loading control. Numbers correspond to the average protein abundance relative to the lane of DMEM. The image is representative of at least three independent replicates.

Lysine acetylation in H-NS has been observed in various proteomics studies (21). To confirm this observation, a pan-acetyllysine antibody was used to analyze the acetylation levels of purified H-NS from *E. piscicida* (EIB202), *S*. Typhimurium (SL1344) and *E. coli* (BL21). The Western blot analysis indicated that the apparent acetylation level of H-NS in *E. piscicida* was similar to that in *S*. Typhimurium and *E. coli* (Figure 1A). Moreover, the degree of H-NS acetylation in DMEM cultures of *E. piscicida* was 2-fold higher than that in LB medium (Figure 1B). Compared to the nutrient-rich LB medium, which is suitable for bacterial cultivation, DMEM is a basal medium with minimal essential nutrition and is commonly used to induce T3/T6SS expression (28). We thus hypothesized that the acetylation of lysine residues in H-NS is associated with the activation of the T3/T6SS.

To further investigate the potential factors affecting the H-NS acetylation level and T3/T6SS activation, synthetic M9 medium and DMEM were exploited as minimal-nutrient and tissue-like media, respectively. When cultured in M9 medium inducing the stringent response (36), H-NS exhibited lower acetylation levels, consistent with the blank secretion profile (Figure 1C, D). With the gradual supplementation of casamino acids in M9 medium, the H-NS acetylation level increased, which correlated with the increased expression of T3/T6SS (Figure 1C, D). Conversely, adding casamino acids to DMEM decreased H-NS acetylation levels and T3/T6SS expression (Figure 1C, D). Based on the inactivated T3/T6SS in LB medium and activated T3/T6SS in DMEM, we speculated that amino acid-free nutrition (M9 medium) inhibits T3/T6SS to save energy as the stringent response; conversely, excessive amino acids (LB medium) restrain T3/T6SS to enable fast growth. Accordingly, the growth rate of bacteria gradually increased from M9 to DMEM with the addition of casamino acids (Figure 1D). Furthermore, an elevation in the lysine acetylation level of H-NS corresponded to an increase in the protein level (Figure 1C, Supplementary Figure S4A). Together, the switch of T3/T6SS depends on the regulation of H-NS acetylation, which is influenced by amino acid nutrition.

Reprogrammed host immune metabolism can produce immune metabolites to modulate microbial pathogenesis during bacterial infection. To explore whether H-NS acetylation is involved in this process, several immune metabolites were utilized to assess the acetylation level of H-NS. In the presence of glucose and citrate, H-NS exhibited higher acetylation levels than in DMEM, while acetyllysines were reduced due to itaconate, fumarate and succinate (Figure 1E). The expression profile of T3/T6SS exhibited consistent trends (Figure 1F), supporting the notion that the H-NS acetylation level modulates microbial pathogenesis.

### Lysine acetylation affects the xenogeneic silencing function of H-NS

**Figure 2.**
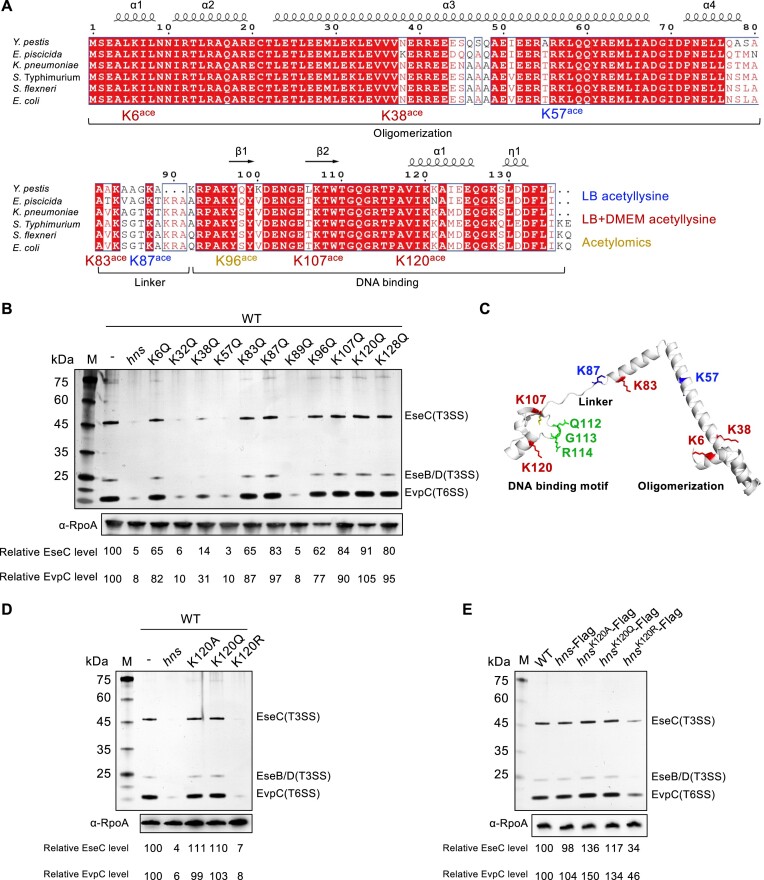
Identification and functional analysis of H-NS acetyllysine. (**A**) Acetylation sites of H-NS in *E. piscicida*. Multiple alignments of H-NS from *Y. pestis* (632), *K. pneumoniae* (HS11286), *S*. Typhimurium (SL1344), *S. flexneri* (623) and *E. coli* (O157:H7). The purified H-NS-His_6_ from *E. piscicida* cultured in LB medium and DMEM was analyzed with LC−MS/MS. Blue represents the acetyllysine site identified in LB medium; orange represents the acetyllysine site identified in a previous acetylomics; red represents the acetyllysine site jointly identified in both LB medium and DMEM. (**B**) Extracellular protein profile analysis of T3SS and T6SS expression in the indicated strains. Different simulated constant acetylation (K-Q) sites were based on the pUT plasmid. The image is representative of at least three independent replicates. (**C**) Structure of H-NS. The structure was predicted using SWISS-MODEL and annotated with PyMOL. (D, E) Extracellular protein profile analysis of T3SS and T6SS expression influenced by K120 acetylation. Different simulations of K120 were based on the pUT plasmid (**D**). Different simulations of K120 were based on *sacB*-based gene editing *in situ* in the genome (**E**). The supernatants from identical amounts of bacteria were resolved by SDS−PAGE, and RpoA was used as a loading control. Numbers correspond to the average protein abundance relative to the leftmost lane. The image is representative of at least three independent replicates.

To identify the specific acetylated lysine residues in H-NS, the H-NS of *E. piscicida* cultured in LB and DMEM was purified and analyzed using mass spectrometry. A total of 8 acetyllysine sites were identified and distributed in the oligomerization domain, DNA binding domain and intermediate flexible linker region (Figure 2A; Supplementary Figure S2). Of them, acetylated K57 and K87 occurred in LB medium. K6, K38, K83, K107 and K120 were identified as acetylated residues in both media. Combining the previously identified acetylated K96 in *E. piscicida* acetylomics (29), the total lysine acetylation sites of *E. piscicida* are indicated by red arrows, while the lysine acetylation sites of *E. coli* are indicated by orange arrows (21) (Supplementary Figure S4D). Both *E. piscicida* and *E. coli* had lysine acetylation at the K6, K57, K83, K87, K96, K107 and K120 sites. Notably, *E. piscicida* had another acetylation at K38, whereas most bacteria, including *E. coli*, had asparagine at that site. Although mass spectrometry identified most acetylated lysine residues, it is possible that the remaining lysines could also be acetylated under specific situations. Therefore, all lysines were mutated to glutamine to mimic constant lysine acetylation, and the corresponding expression profiles of T3/T6SS were determined (Figure 2B). Compared to the overexpression of wild-type H-NS, the variants with acetylated residues identified in DMEM, including K6Q, K83Q, K87Q, K96Q, K107Q, K120Q and K128Q, failed to inhibit T3/T6SS expression. In particular, the inhibition ability of K120Q almost entirely disappeared, with 91% relative EseC and 105% relative EvpC expression levels (Figure 2B).

Specifically, the green loop represents the conserved binding motif ‘QGR’, which is inserted into the narrow minor groove, and the side chain of K120 aligns with the orientation of the green loop (Figure 2C and Supplementary Figure S5). Riccardi *et al.* demonstrated the probability of finding DNA within 0.6 nm of an H-NS protein residue, with R114 having the highest probability, followed by G113, Q112, R93 and K120 (37). We initially obtained 2000 homologous sequences of *E. piscicida* H-NS using PSI-BLAS, which were distributed across 385 genera/species. Multiple sequence alignment indicated that K120 is a relatively conserved amino acid (Supplementary Figure S4D). We then engineered the variants K120A and K120R to simulate inactivation and constant nonacetylation, respectively. Compared to K120Q, K120A also lost its ability to inhibit T3/T6SS, while K120R inhibited T3/T6SS expression (Figure 2D). To further validate the influence of K120 on T3/T6SS expression, we generated three *in situ* mutations simulating inactivation (H-NS^K120A^), constant nonacetylation (H-NS^K120R^) and constant acetylation (H-NS^K120Q^) and fused them to a Flag tag for further ChIP experiments (Figure 2E). The H-NS^K120A^ and H-NS^K120Q^ variants lost the ability to inhibit T3/T6SS, while H-NS^K120R^ maintained the inhibitory function of wild-type H-NS. In conclusion, K120 is proposed to be a crucial acetylation site for the xenogeneic silencing of H-NS.

### Acetylation of H-NS^K120^ impairs DNA binding ability *in vitro* and *in vivo*

**Figure 3.**
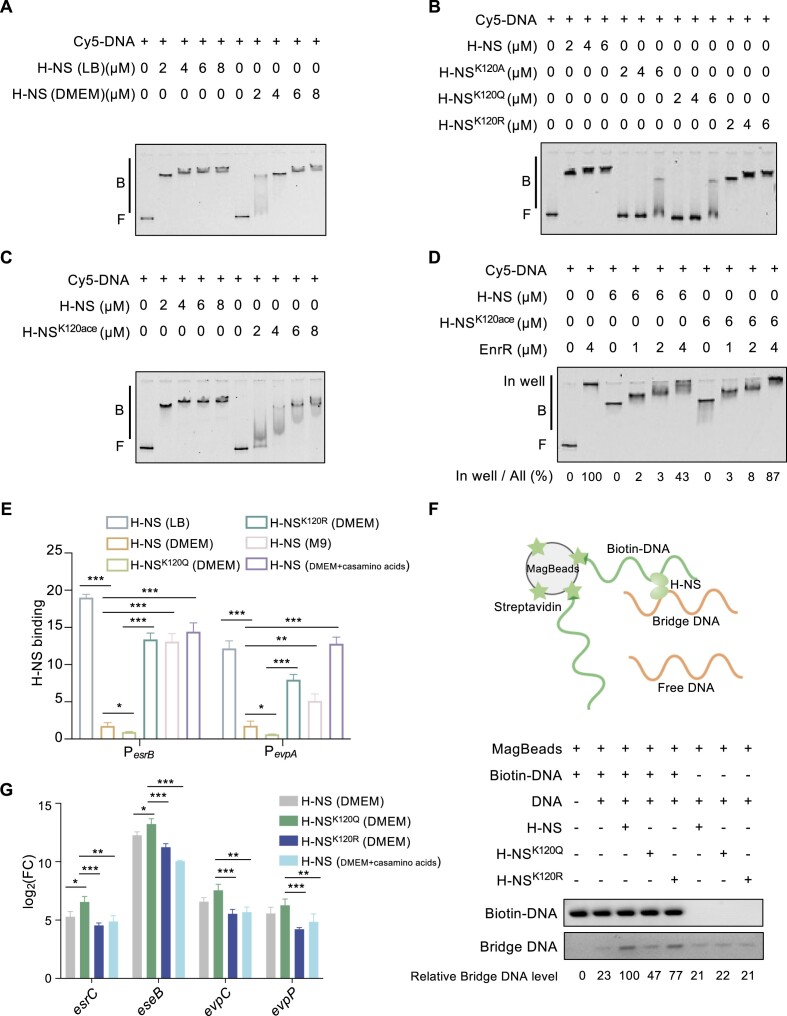
Acetylation of K120 reduces the DNA binding ability of H-NS. (**A**) EMSAs of the DNA-binding abilities of H-NS purified from *E. piscicida* cultured in LB medium and DMEM. H-NS mixed with 20 ng of Cy5-labeled P*_esrB_* probe was added to the EMSA reactions. (**B**) EMSAs of the DNA-binding abilities of H-NS and the H-NS^K120^ mutant purified from BL21(DE3). (**C**) EMSA of the DNA-binding abilities of H-NS and H-NS^K120ace^ purified from BL21(DE3). (**D**) EMSA of the competition between EnrR and H-NS or H-NS^K120ace^ for binding to P*_esrB_* DNA *in vitro*. All images are representative of three independent experiments. (**E**) *In vivo* binding of H-NS and mutants to the promoter regions of *esrB* and *evpA*. The proteins H-NS, H-NS^K120Q^, and H-NS^K120R^ were purified from *E. piscicida* grown in LB medium, DMEM and DMEM + Casamino acids for 14 h using ChIP with anti-Flag magnetic beads. The relative enrichment of H-NS variants on P*_esrB_* and P*_evpA_* was assayed by qPCR with primers targeting P*_esrB_* and P*_evpA_*. The relative H-NS binding enrichment was normalized to that of *dnaA*. (**F**) A DNA pull-down bridging assay detected the bridging ability of H-NS. Streptavidin-coated magnetic beads bound to biotinylated DNA (500 bp) were incubated with unlabeled DNA and H-NS variants. The bridged DNA was then amplified and resolved on an agarose gel. The image is representative of at least three independent replicates. (**G**) Relative transcripts of representative T3/T6SS genes in the indicated strains as assessed by qRT−PCR. The *dnaA* gene was used as an internal control. *** *P* < 0.001; ** *P* < 0.01; * *P* < 0.05; N.S., *P* > 0.05 based on Student's *t* test.

Next, an electrophoretic mobility shift assay (EMSA) was used to investigate the impact of lysine acetylation on the ability of H-NS to bind DNA. Initially, the T3/T6SS core regulatory promoter P*_esrB_* was labeled with Cy5 and incubated with H-NS purified from *E. piscicida* cultured in DMEM and LB medium. As expected, H-NS from LB medium bound to all labeled DNA at a concentration of 2 μM, while H-NS from DMEM did not (Figure 3A). Given that H-NS from these media have differences not only in acetylation, we further compared the influence on DNA binding ability by H-NS acetylation (Figure 3B). The H-NS mutants with inactivation and constant lysine acetylation exhibited significantly impaired DNA binding ability for P*_esrB_* compared to wild-type H-NS, while the nonacetylation mutant K120R exhibited similar binding profiles as wild-type H-NS (Figure 3B).

Our attempts to mimic acetyllysine using other amino acids could not entirely represent natural acetyllysine; consequently, a site-specific acetylated protein expression system was employed to achieve full acetylation of the H-NS^K120^ protein (Supplementary Figure S4C) (17,31). Although P*_esrB_* was bound by H-NS^K120ace^, the DNA-binding affinity of H-NS^K120ace^ was apparently weaker than that of H-NS, with relatively diffuse binding bands (Figure 3C). The diffuse binding bands of H-NS^K120A^, H-NS^K120Q^ and H-NS^K120ace^ indicated the impaired DNA-binding ability of H-NS due to acetylation at K120.

Furthermore, the presence of EnrR was used to examine the DNA-binding ability of H-NS^K120^ because EnrR can expel H-NS to compete for binding to P*_esrB_*(9). EnrR can bind to AT-rich DNA sequences with unique interactions with both major and minor grooves. EMSA indicated that the EnrR-P*_esrB_* complex migrated to the well position, suggesting a strong DNA-binding ability (13) (Figure 3D). However, with diffuse bands, P*_esrB_* was completely bound by a constant amount of H-NS or H-NS^K120ace^ (Figure 3D). In the presence of EnrR, protein−DNA complexes migrated toward the well, and the shifts were enhanced by the titrated concentrations of EnrR. The ratio of protein−DNA complexes in each well divided by all protein−DNA complexes was calculated. When the EnrR concentration was 4 μM, H-NS binding DNA was competitively reduced by 43%, while 87% H-NS^K120ace^ was expelled by EnrR (Figure 3D), indicating a weaker DNA-binding ability of H-NS^K120ace^ than that of H-NS.

To investigate the impact of H-NS acetylation on the *in vivo* binding of T3/T6SS clusters, ChIP−qPCR was utilized to quantify the enrichment of P*_esrB_* and P*_evpA_* by H-NS variants (Figure 3E). Compared to H-NS-bound DNA in LB medium, the ability of H-NS to bind to the P*_esrB_* and P*_evpA_* promoters significantly decreased in DMEM. Moreover, H-NS^K120Q^ cultured in DMEM exhibited a further reduction in binding. However, H-NS^K120R^ cultured in DMEM showed greater binding affinity for the T3/T6SS promoter than wild-type H-NS and H-NS^K120Q^ in DMEM. In the presence of casamino acids, the H-NS bound to P*_esrB_* and P*_evpA_* promoters significantly accumulated.

H-NS has been proposed to modulate gene regulation through two different binding modes: the stiffening mode and the bridging mode (22). Therefore, a modified DNA pull-down bridging assay was further performed to explore the influence of H-NS acetylation on its DNA bridging capacity. The presence of H-NS generated the apparent bridged DNA, while H-NS^K120Q^ could not fully recover the bridged DNA, indicating that acetylation affects the bridging ability of H-NS (Figure 3F). Moreover, qRT−PCR assays revealed that the transcription level of several T3/T6SS genes controlled by P*_esrB_* and P*_evpA_* exhibited similar tendencies as the corresponding enrichment by H-NS variants (Figure 3G). The acetylation of H-NS enhanced the transcript levels of *esrC*, *eseB*, *evpC* and *evpP* (H-NS^K120Q^ versus H-NS^K120R^/H-NS), which is consistent with the DNA-binding ability of H-NS^K120Q^ being the lowest, as shown in Figure 3E. In summary, these findings demonstrated that H-NS acetylation modulates its DNA binding ability for T3/T6SS clusters *in vivo* and *in vitro*.

### Lysine acetylation reduces the DNA-binding ability of H-NS in global AT-rich regions

**Figure 4.**
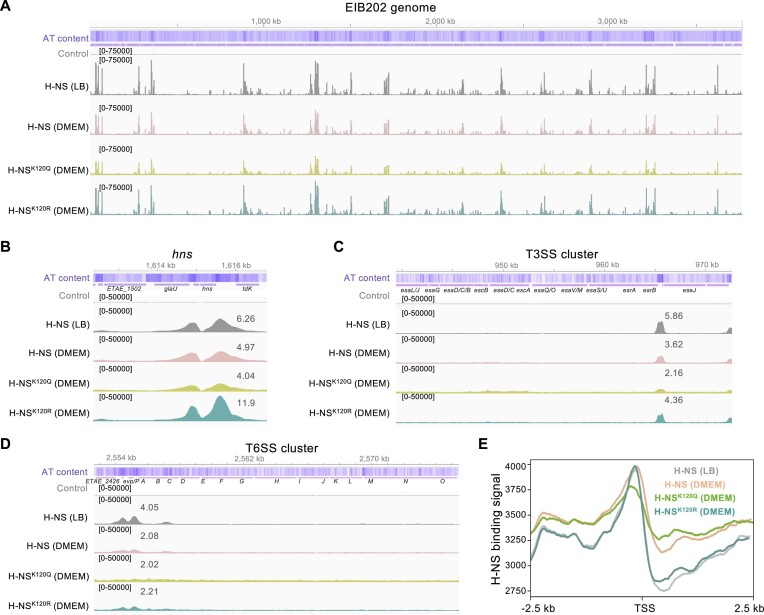
Acetylation of K120 affects the binding of H-NS to the AT-rich DNA region. (**A**) Overview of the genome information and ChIP-seq mapping results. The AT content was calculated using a 100 bp window, with purple representing AT-rich areas. The ChIP-seq results for the control and four H-NS variants are shown, where the peak height (y-axis) represents the sequencing read depth at each genomic position (x-axis). The data are a merge of two replicates. (**B–D**) The identified H-NS binding sites over the *hns* gene (B) and GIs of T3/T6SS (C, D). The recruitment levels of binding sites around the promoter region of the indicated genes are shown. (**E**) Statistical analysis of the H-NS binding signal at transcriptional initiation sites across the genome. Python deepTools2 computeMatrix and plotHeatmap tools were used with input H-NS ChIP-seq bigwig files and EIB202 transcriptional initiation site bed files.

To fully understand the impact of lysine acetylation on the global DNA-binding profiles of H-NS, high-throughput sequencing of the ChIP DNA fragments was conducted using different H-NS variants. The overall DNA-binding peaks of all H-NS variants were consistent and leaned toward the AT-rich region (Figure 4A), consistent with the known binding characteristics of H-NS (3,4). Across the board, the binding peaks of H-NS purified from *E. piscicida* cultured in LB medium were much stronger than those in DMEM, and the binding strength of H-NS^K120Q^ (DMEM) was the weakest. The constant nonacetylation of K120R rescued the binding strength of H-NS to the level of H-NS in LB medium (Figure 4A).

Upon closer inspection, the specific binding peaks still exhibited a similar trend, with a preference for AT-rich regions. The recruitment levels are labeled on the peaks, with H-NS (LB) and H-NS^K120R^ (DMEM) showing the strongest binding peaks (Figure 4B–D). Furthermore, the protein abundance of H-NS varied among the different H-NS variants, of which H-NS^K120R^ (DMEM) exhibited the lowest level and H-NS^K120Q^ (DMEM) possessed the highest level (Supplementary Figure S4B). Correspondingly, H-NS^K120Q^ (DMEM) exhibited the lowest recruitment level at the *hns* promoter, while H-NS^K120R^ (DMEM) showed the highest recruitment level at the *hns* promoter (Figure 4B). H-NS is a self-regulating protein, and there were two binding peaks found in the head and tail of the *hns* gene (Figure 4B). These findings indicate that lysine acetylation on H-NS affects its self-regulation, which enhances *hns* expression. In the T3SS cluster, the only concentrated peak was located in the promoter of *esrB* (Figure 4C). Similarly, the promoters P*_evpP_* and P*_evpA_* were tightly bound by H-NS in the T6SS cluster (Figure 4D).

Furthermore, a statistical analysis of the H-NS binding signal at transcriptional initiation sites across the genome was performed using deepTools2 (34). The combined signal peaks of the four groups were located adjacent to the left of the transcription start site (TSS), reflecting the binding of H-NS in the regulatory regions of transcriptional initiation (Figure 4E; Supplementary Figure S3). Similar to the observed differences in overall H-NS binding peaks in Figure 4A, H-NS^K120Q^ showed the weakest binding signal at the TSS, while the binding signal of H-NS^K120Q^ at non-TSS locations was the most robust (Figure 4E, light green line). These findings demonstrated that the binding capacity of H-NS to AT-rich regions decreases due to lysine acetylation.

### Acetylation of K120 leads to impaired possession of AT-rich DNA

**Figure 5.**
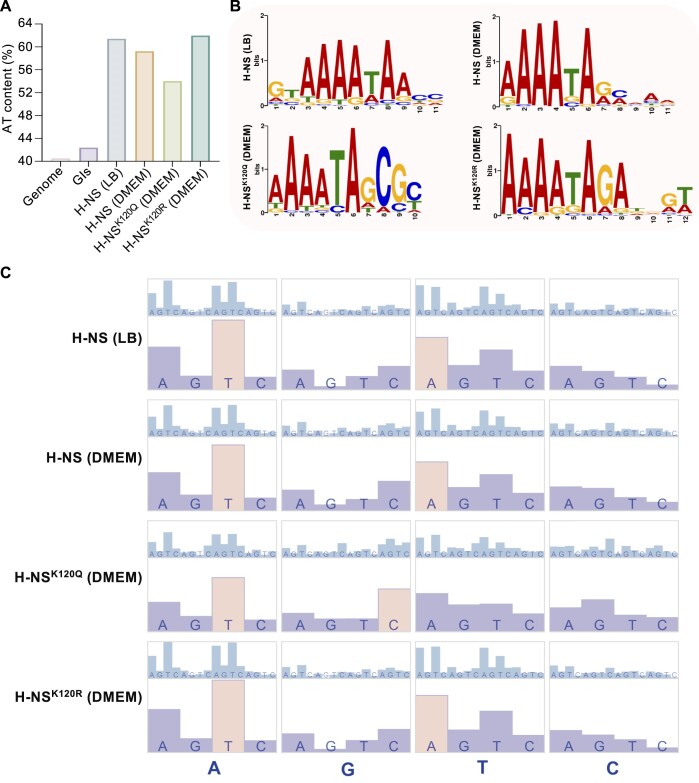
Acetylation of K120 impairs the AT DNA recognition ability of H-NS. (**A**) AT% content of ChIP-seq peaks compared to the averages of the GIs and the genome. (**B**) H-NS-binding motifs derived from STREME analysis of the ChIP-seq peaks. The conserved binding sequence of H-NS (LB) is ‘GTAAAATAA,’ H-NS (DMEM) is ‘AAAATAGC’, H-NS^K120Q^ (DMEM) is ‘AAAATAGCGC’ and H-NS^K120R^ (DMEM) is ‘AAAATAGA’. (**C**) Dinucleotide and trinucleotide preference statistics for ChIP peaks. The frequencies of 16 dinucleotides and 64 trinucleotides in ChIP-seq peaks were calculated and divided by 0.0625 and 0.015625, respectively. Orange represents the two most significantly bound dinucleotides. Scale bars: 0.5–2 (dinucleotide), 0.4–2.7 (trinucleotide). The image was generated using Advanced Circos in TBtools.

Was the peak for acetylated H-NS^K120^ binding DNA lower than that for the nonacetylated form because this protein does not like to bind to AT-rich DNA? To answer this question, we further analyzed ChIP-seq peaks of H-NS variants using MACS2 (Supplementary Table S3). The average AT content of the *E. piscicida* genome is 40.3%, and GIs have a slightly elevated AT content of 42.4% (26). The AT ratio of H-NS (LB) binding sites was 61.4%, confirming the preference of H-NS for binding to AT-rich DNA. In contrast, H-NS^K120Q^ had an AT ratio of only 54% (Figure 5A). Next, the STREME algorithm was exploited to determine the consensus H-NS binding sequences (32). The consensus binding sequence of H-NS^K120Q^ was 5′-AAAATAGCGC-3′ (*E* < 0.05), indicating a reduced preference for AT-rich sequences compared to the other three groups (Figure 5B). Subsequently, the proportions of dinucleotides (16 types) and trinucleotides (64 types) were analyzed. Except for H-NS^K120Q^ (DMEM), which primarily preferred AT (9.979%) and GC (8.542%) dinucleotides, the other three groups exhibited a preference for AT and TA dinucleotides (Figure 5C). These analyses suggested that acetylation of K120 reduces the binding of H-NS to AT-rich DNA fragments.

**Figure 6.**
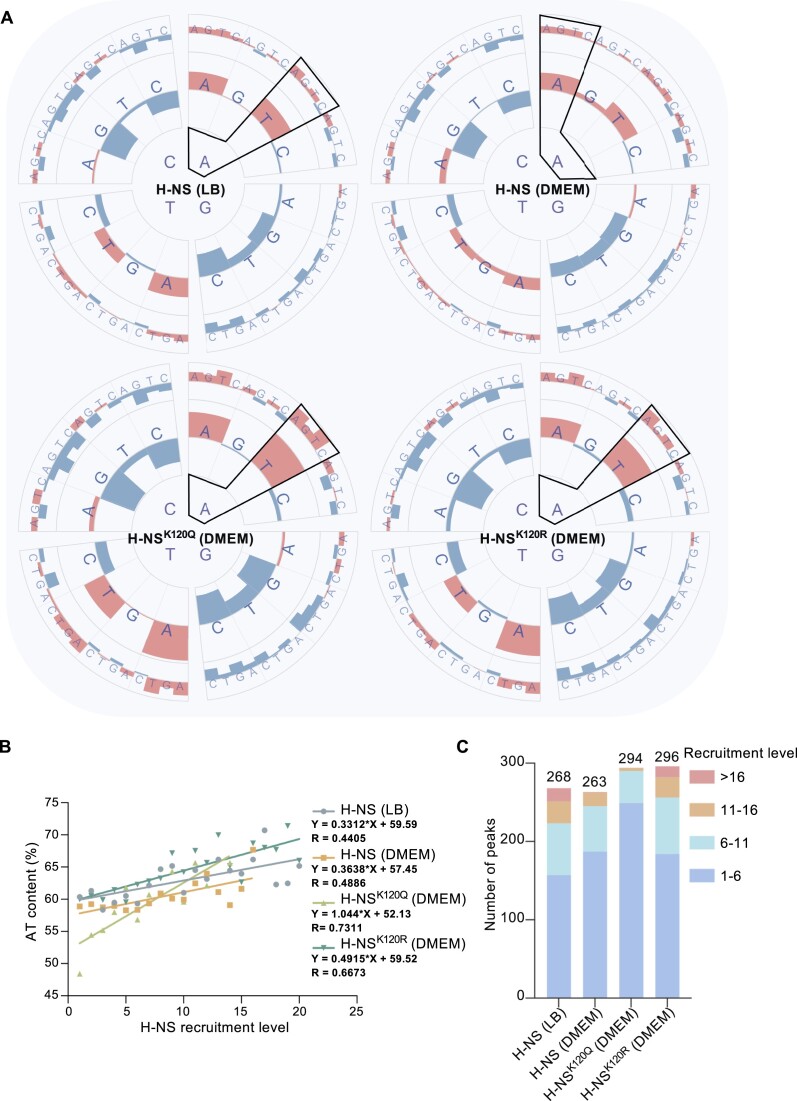
Acetylation of K120 impairs the AT-rich DNA recognition ability of H-NS. (**A**) The height of red/blue bars represents the positive/negative coefficient (Spearman's rho) between the oligonucleotide usage deviations and H-NS recruitment levels in ChIP-seq peaks. Bar scales: −0.52–0.52 (dinucleotide and trinucleotide). The image was generated using Advanced Circos of TBtools. (**B**) Relationship between averaged AT% values (Y-axis) of pooled ChIP-seq peaks (with 100 bp central region) and mean recruitment levels (Y-axis) determined in the ChIP-seq analysis. The recruitment level dataset was divided into 20 ranges. (**C**) Statistics on the number of ChIP peaks and peaks with different recruitment levels.

Furthermore, we examined the relationship between the oligonucleotide composition (dinucleotide, trinucleotide) of the abundant peaks and recruitment levels (9,33) (Figure 6A). The strongest positive correlation between H-NS (LB) binding and dinucleotide was with AT (Spearman's rho = 0.296; *P* = 8.20912E-07), while the strongest negative correlation was with CG (Spearman's rho = −0.368; *P* = 5.34627E-10). Although H-NS^K120Q^ had the weakest AT-rich DNA binding ability, the correlation between the AT (Spearman's rho = 0.517; *P* = 1.4881E-21) or GC (Spearman's rho = −0.500; *P* = 5.00716E-20) dinucleotide and the recruitment levels of H-NS^K120Q^ were the highest, which somewhat contradicts the low AT-rich DNA binding capacity.

We then analyzed the relationship between the average AT% values of ChIP-seq peaks and recruitment levels. With low recruitment levels (Fc: 1∼6) of H-NS^K120Q^, the average AT% content was less than 55% (Figure 6B), and the relative proportion of low recruitment level fragments to the total fragments was 249/294 (Figure 6C). A positive correlation between AT% and the protein recruitment level of H-NS was identified, and the correlation coefficient of AT% and H-NS was enhanced along with increased acetylation of H-NS (1.044 of H-NS^K120Q^ vs. 0.4915 of H-NS^K120R^) (Figure 6B). H-NS^K120Q^ cannot efficiently compete with countersilencing proteins for binding to AT-rich DNA; in other words, the acetylation of K120 in H-NS leads to impaired possession of AT-rich DNA. H-NS^K120Q^ (DMEM) exhibited fewer binding events at the TSS but had the highest intracellular protein level, suggesting that excessive H-NS^K120Q^ protein binds to numerous non-TSS fragments (Figure 4E and Supplementary Figure S4B). When the call peaks of H-NS^K120Q^ (DMEM) were compared with those of H-NS (DMEM) and H-NS^K120R^ (DMEM), 86 and 74 peaks were obtained, respectively (Supplementary Table S3). The recruitment level values of these peaks were low, at approximately 1.5-fold. The AT content was calculated at the highest peak positions in both groups, resulting in 45.42% and 43.53%, respectively (Supplementary Table S3 and Supplementary Figure S6).

### Disrupting H-NS acetylation attenuates bacterial virulence

**Figure 7.**
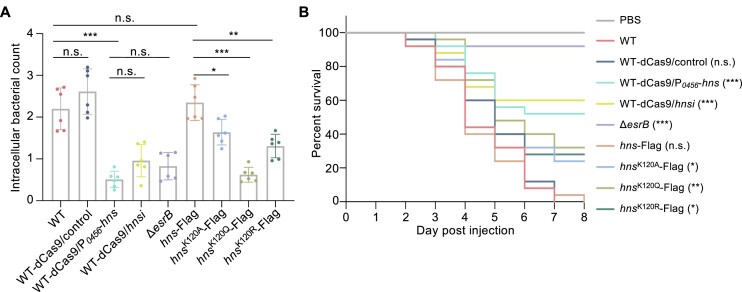
Lysine acetylation of H-NS modulates bacterial virulence in cell and zebrafish models. (**A**) J774A.1 cells were infected with the indicated strains at an MOI of 10. After 2 h of infection, Opti-MEM was added to cell cultures with 50 μg/ml Gm to kill extracellular bacteria, and the cells were incubated at 35°C with 5% CO_2_ for another 2 h. Then, the cultures were treated for 30 min with PBS containing 1% Triton X-100 to disrupt the cells. Intracellular bacteria were enumerated by serial dilution plating on LB plates. *** *P* < 0.001; ** *P* < 0.01; * *P* < 0.05; N.S., *P* > 0.05 based on Student's *t* test. (**B**) Survival curve of zebrafish challenged with the indicated strains. A total of 400 CFU of bacteria suspended in 5 μl of PBS was intramuscularly injected into each zebrafish; PBS was used as the negative control. A total of 25 zebrafish were injected with each strain, and mortality was monitored quarterly daily. ** *P* < 0.01; *** *P* < 0.001 based on Kaplan−Meier survival analysis with a log-rank test (Mantel−Cox).

To characterize the role of H-NS acetyllysine in regulating virulence, macrophage J774A.1 and zebrafish infection models were established (Figure 7A, B). Consistent with our previous data, the virulence of Δ*esrB* was greatly weakened, which led to poor colonization in macrophages and failure to cause lethality in zebrafish within 8 days (30). Knockdown and overexpression of *hns* both resulted in defects in macrophage colonization and reduced lethality in zebrafish, which were also observed in H-NS variants. These findings indicated that the inhibition of T3/T6SS by H-NS is a coordinated strategy and that the occurrence and removal of K120 acetylation serve as a switch for turning on and off the inhibition mediated by H-NS.

## Discussion

**Figure 8.**
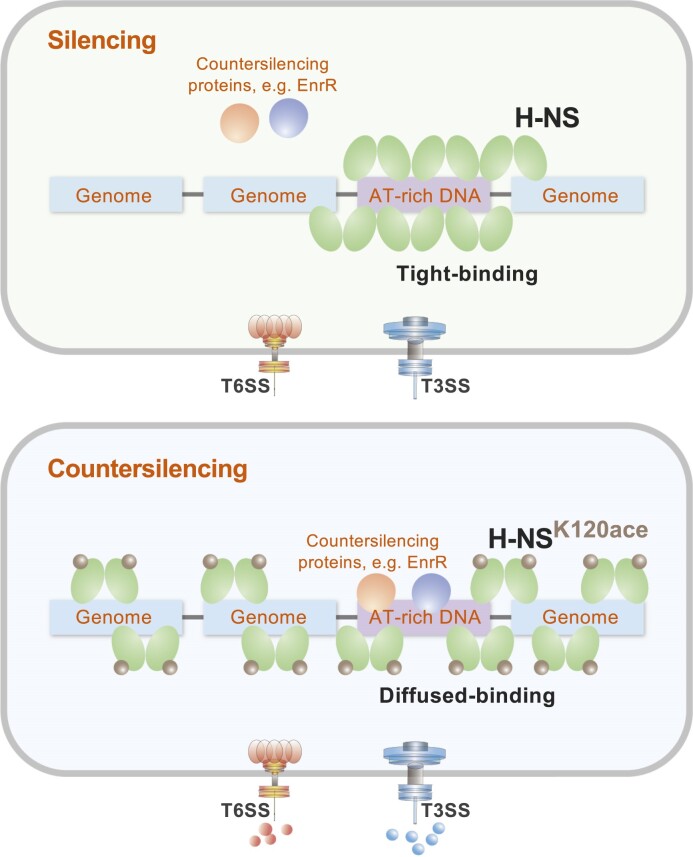
The proposed model of acetylation regulating the AT-rich DNA possession ability of H-NS. Initially, H-NS prefers to interact with AT-rich DNA regions in the genome, with tight binding and robust occupancy. This interaction obstructs the transcription process of RNA polymerase and ultimately silences the expression of heterologous genes. However, upon acetylation of H-NS^K120^, its binding to AT-rich DNA regions becomes diffuse. As a result, the possession ability of H-NS is reduced, resulting in increased expression of heterologous downstream genes.

Analogous to eukaryotic histones, bacterial nucleoid-associated proteins undergo posttranslational modifications at different growth and infection phases (22). More commonly, these modifications occur in response to environmental cues. Here, in *E. piscicida*, H-NS is a lysine-acetylated protein, and the acetylation of H-NS attenuated its ability to silence horizontally transferred genes in response to amino acid nutrition and immune metabolites. LC−MS/MS analysis identified eight acetyllysine sites in H-NS. The specific acetylation site K120, located in the DNA binding domain of H-NS, modulated its DNA-binding ability and AT-rich DNA recognition ability. Specifically, the number of H-NS^K120Q^ binding peaks was not reduced, but there was a significant decrease in the possession of AT-rich DNA by H-NS^K120Q^ (Figure 8).

The importance of amino acid nutrition in bacterial colonization within the host has been demonstrated (38). Our previous Tn-seq data indicated that mutants of disrupted genes involved in amino acid metabolism and transport were quickly eliminated in the host (27). Free-living *E. piscicida* resides in nutrition-deficient water, similar to M9 medium, and is trapped in the stringent response, such as low acetylation levels of H-NS inhibiting T3/T6SS (25,36). Upon invading the host, it encounters a relatively nutrient-rich environment compared to water, similar to DMEM (30,38,39). The acquisition of amino acids from the host enhances the acetylation level of H-NS, relieving the inhibition of T3/T6SS to disrupt the host immune system and facilitate systemic infection. Toward the end of the infection, the ample amino acid nutrition from the moribund host, similar to LB medium, switches H-NS to a low acetylation level again, which inhibits T3/T6SS and allows the bacteria to focus energy on growth and reproduction.

Bacterial infections can induce host immune metabolism reprogramming, which serves as both an immune defense response and a signal modulating bacterial virulence (40,41). *S*. Typhimurium can sense succinate to promote antimicrobial resistance and T3SS secretion (40). The accumulation of itaconate leads to decreased lipopolysaccharide production and decreased acetylation levels of CspC in *P. aeruginosa* (42,43). During infection, *E. piscicida* manipulates host arginine metabolism to interfere with inflammation occurrence (44). In the presence of itaconate, succinate, or fumarate, the acetylation level of H-NS and T3/T6SS expression were reduced (Figure 1E, F). We speculated that bacteria sense amino acids and immune metabolism to modulate the acetylation status of regulatory proteins, such as H-NS of *E. piscicida*, enabling them to evade immune clearance.

As a rapid and reversible posttranslational modification, lysine acetylation allows bacteria to adapt quickly to the external environment, and crosstalk between lysine acetylation and metabolism and virulence has been established (14–17,23,45,46). H-NS is a polylysine protein with relatively conserved lysine positions (Figure 2A). The N-terminus of H-NS is involved in sensing temperature, salinity and other signals to adjust the oligomerization state (11,47). The linker region of H-NS also plays an important role in maintaining its function, and it has a flexible structure (48). The identified acetyllysine K83 and K87 in the linker region resulted in the loss of H-NS inhibitory function on T3/T6SS (Figure 2B). Lysine acetylation in the DNA binding domain modulated the inhibition of HGT of the GIs of T3/T6SS (Figure 2B). This was primarily due to the effect of electric charge neutralization through acetylation, which impairs the ability of lysine to bind negatively charged DNA (46). The specific consequences and functions of lysine acetylation at different positions of H-NS, as well as how they affect the conformation of H-NS, require further study.

The structure of the DNA binding domain of H-NS in *Salmonella* was solved with a high quality solution (4). The amino acid sequence of *E. piscicida* H-NS is highly similar to that of *Salmonella*, and the predicted DNA binding domain structure of *E. piscicida* H-NS is also similar to that of *Salmonella* (Figure 2A; Supplementary Figure S5C). Both structures consist of a two-stranded antiparallel β-sheet (β1 residues 97–100, β2 residues 105–109), an α-helix (residues 117–126), and a 3_10_ helix (residues 130–133). The conserved binding site ‘QGR’, which interacts with the narrow minor groove, is located on the loop between the β2-sheet and α-helix, and K120 is located on the α-helix (4). The data from different research groups regarding the interaction between the H-NS DNA binding domain and DNA indicate that the most critical amino acids for DNA binding are ‘QGRTPA’ at residues 112–117, with K96, K120 and K121 possibly involved in DNA binding as well (4,37). Moreover, other xenogeneic silencers, such as MvaT and Rok, recognize the DNA minor groove through residues ‘R-GN’ and ‘N-T-R’, respectively, assisted by lysine residues interacting with the phosphate groups (49,50). However, no direct solved structural evidence of H-NS lysine residues participating in DNA binding exists. It is hypothesized that positively charged lysine residues may play a role during the initial stage of H-NS moving closer to the DNA (37).

DNA that can undergo HGT typically exhibits an AT-rich feature, similar to the −10 to −35 region that influences the normal transcription of RNAP (51,52). Most bacteria possess H-NS or analogous proteins, such as Lsr2 in *Mycobacterium tuberculosis*, MvaT in *P. aeruginosa*, and RoK in *Bacillus subtilis*, to regulate transcription by recognizing the narrow minor groove of AT-rich DNA (4,5,53). The clustering of adenine residues narrows the minor groove and increases the negative charge, which is specifically bound by positively charged amino acids such as arginine and lysine (51,54). Acetylation of lysine leads to the loss of a positive charge; therefore, acetylation of K120 reduced the AT-rich DNA-binding preference of H-NS (Figures 5 and 6). The highest recruitment levels were only achieved when acetylated H-NS at K120 bound to DNA with relatively high AT%.

H-NS acts as a protein scaffold for maintaining genome structure and regulating gene expression, similar to eukaryotic histones (47,55). However, unlike histones, H-NS primarily silences AT-rich regions associated with HGT (2). HGT genes typically possess a high AT content and H-NS tends explicitly to bind to AT-rich sequences. While most transcriptional regulatory proteins and sigma factors enhance transcription by binding to AT-rich DNA, H-NS silences it by exclusively occupying these regions without sharing them with other DNA-binding proteins (56). Bacteria have developed a series of countersilencing mechanisms (1,10). Countersilencing proteins, such as EnrR, which we previously discovered (9), recognize AT-rich DNA and use their N-terminal α-helix to displace H-NS from the promoter regions of the virulence GIs, congruent with the function of PhoP and HlyA in *S*. Typhimurium (6), LuxR and HlyU in *Vibrio* (7,57), and VirB in *Shigella* (58).

The alternate mechanism relies on changes in the properties of H-NS. Many previous studies on the properties of H-NS have focused more on the changes in H-NS conformation caused by physical and chemical factors such as osmotic pressure and temperature (11,59). Few studies have revealed that various H-NS PTMs affect the inhibition of AT-rich genes. Phosphorylation of T13 in *S*. Typhimurium H-NS reduces its dimerization and weakens its DNA binding affinity (12), while phosphorylation of S42 in *Shewanella* H-NS responds to temperature changes and regulates prophage excision (13). In summary, lysine acetylation, as a type of PTM, affects the possession of AT-rich DNA by H-NS, which leads to the expression of HGT and plays a crucial role in the transition between H-NS silencing and desilencing of HGT.

## Supplementary Material

gkad1172_supplemental_files

## Data Availability

The ChIP-seq data has been deposited in BioProject (https://www.ncbi.nlm.nih.gov/bioproject) under the accession number PRJNA994976. Mass spectrometry data has been deposited in PRIDE (https://www.ebi.ac.uk/pride/) with identifier PXD047152.
