# Supplementary material for: Lysine acetylation regulates the AT-rich DNA possession ability of H-NS
Source: Nucleic Acids Res. 2023 Dec 7;52(4):1645–60. doi: 10.1093/nar/gkad1172 (PMC10899749; doi:10.1093/nar/gkad1172)
Supplement: gkad1172_supplemental_files [file gkad1172_supplemental_files.zip › SM data.pdf]

Lysine acetylation regulates the AT-rich DNA possession ability of H-NS

Yabo Liu[1], Mengqing Zhou[1], Yifan Bu[1], Liang Qin[4], Yuanxing Zhang[2, 3], Shuai Shao[1, 2, 3, *], Qiyao Wang[1, 2, 3]

[1] State Key Laboratory of Bioreactor Engineering, East China University of Science and Technology, Shanghai 200237, China; [2] Shanghai Engineering Research Center of Maricultured Animal Vaccines, Shanghai 200237, China; [3] Laboratory of Aquatic Animal Diseases of MOA, Shanghai 200237, China. [4] New Product R&D, GenScript Biotech Corporation, Nanjing 211100, China

Correspondence: shaoscott@ecust.edu.cn

SUPPLEMENTARY DATA

Figure S1. H-NS participates in regulating T3/T6SS in *E. piscicida* EIB202. (**A**) Distribution of transposon insertion sites around the *hns* gene. A high-density transposon insertion library was generated in *E. piscicida* EIB202 using a mariner-based Himar1 transposon. The genomic distribution of insertion sites was determined using high-throughput sequencing. (**B**) qRT–PCR assays for the indicated transcripts in the WT, WT-dCas9, WT-dCas9/P_{0456}-hns, and WT-dcas9/*hnsi* strains. Strains were grown statically in DMEM at 30°C for 14 h. The presented results are the means ± S.D. (n = 3) relative to WT. ***, $P < 0.001$; **, $P < 0.01$; *, $P < 0.05$; N.S., $P > 0.05$ based on Student's *t* test. *dnaA* was used as the internal control. (**C**) Western blot analysis of EseB and EvpP levels in the indicated strains. A blot of RpoA was used as a loading control. (**D**) Extracellular protein profile analysis of T3SS and T6SS expression in the indicated strains. The supernatant was collected for SDS–PAGE and silver staining. A blot of RpoA was used as a loading control. The image is representative of at least three independent replicates.

Figure S2. Mass spectrometry b/y ionogram of H-NS acetyllysine peptides. The H-NS-His_6 protein was purified from *E. piscicida* cultured in LB medium and DMEM and then subjected to LC–MS/MS analysis. Based on a 42 Da increase, seven acetyllysine sites were identified in the LB medium group and five in the DMEM group.

Figure S3. Statistical analysis of total H-NS binding signals at transcriptional initiation sites throughout the genome. Using Python deepTools computeMatrix and plotHeatmap

functions, H-NS ChIP bigwig files and EIB202 transcriptional initiation site bed files were input.

Figure S4. Characteristics of H-NS lysine acetylation. (A) Alterations in H-NS protein levels in response to amino acid nutrition. Casamino acids were added in a gradient to increase amino acid nutrition in the M9 medium and DMEM. A blot of RpoA was used as a loading control. (B) Alterations in H-NS protein levels in different H-NS variants. (C) Detection of lysine acetylation levels in H-NS and H-NS^{K120ace}. A blot of H-NS-His was used as a loading control. The image is representative of at least three independent replicates. (D) Alignment of H-NS sequences. Similar sequences were searched using three iterations of PSI-BLAST on the NCBI web server with the following parameters: expected threshold = 0.001, max target sequences = 1000 for the first iteration, and 2000 for the remaining two, while other parameters were left as default. The resulting 2000 sequences were used to generate multiple sequence alignment (MSA) using ClustalW, and a consensus sequence was generated using WebLogo3. Lysine acetylation sites of *E. piscicida* are denoted by red arrows, and lysine acetylation sites of *E. coli* are denoted by orange arrows.

Figure S5. Structural superposition of *E. piscicida* H-NS with resolved H-NS structures. (A) Overall structural comparison of *E. piscicida* H-NS. The structure was predicted using SWISS-MODEL and compared with resolved N-terminal (3NR7) and C-terminal (2L93) structures of *Salmonella*. (B) Detailed comparison of the N-terminal region. (C) Detailed comparison of the C-terminal region. Green highlights the conserved binding motif “QGR.”

Figure S6. Identified H-NS^{K120Q} (DMEM) binding sites at non-AT-rich regions.

Table S1. The bacterial strains, plasmids, and primers used in this study.

Table S2. LC–MS/MS peptide information of H-NS in this study.

Table S3. Information on ChIP-seq peaks calculated in this study.

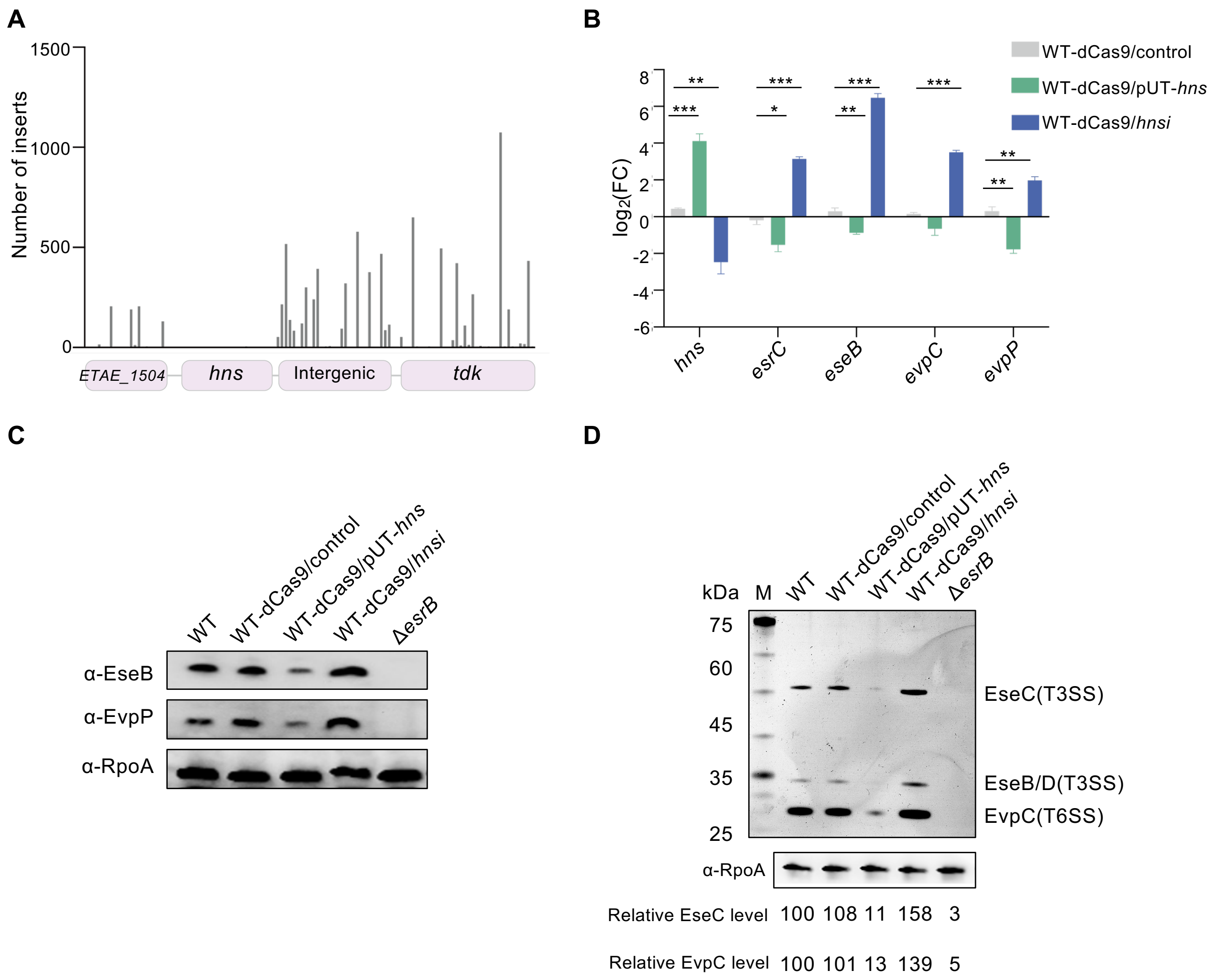
A
1500
1000
500
0
Number of inserts
ETAE_1504
hns
Intergenic
tdk
B
WT-dCas9/control
WT-dCas9/pUT-hns
WT-dCas9/hnsi
log2(FC)
hns
esrC
eseB
evpC
evpP
C
WT
WT-dCas9/control
WT-dCas9/pUT-hns
WT-dCas9/hnsi
ΔesrB
α-EseB
α-EvpP
α-RpoA
D
kDa
M
75
60
45
35
25
EseC(T3SS)
EseB/D(T3SS)
EvpC(T6SS)
α-RpoA
Relative EseC level 100 108 11 158 3
Relative EvpC level 100 101 13 139 5

Figure S1

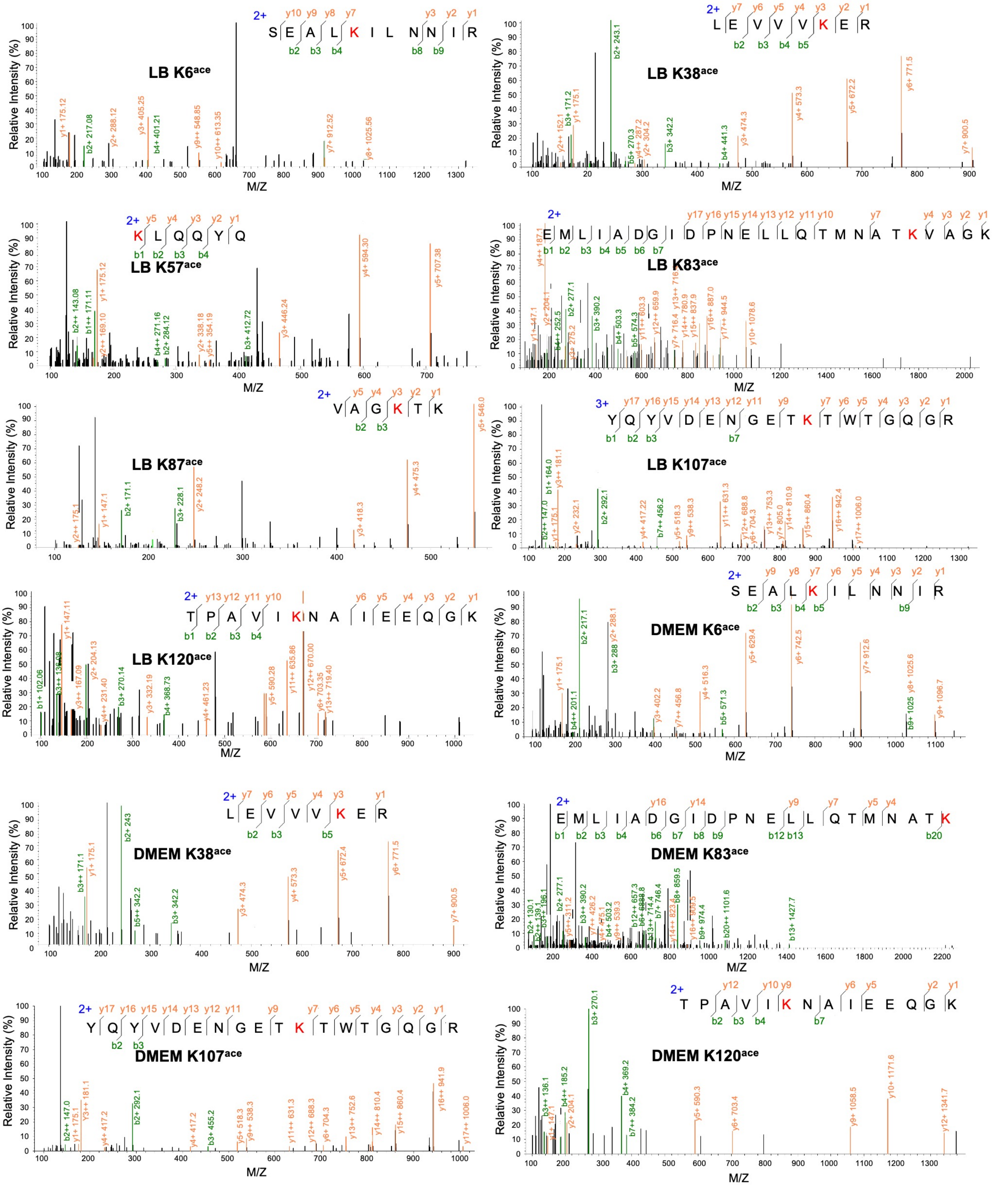
LB K6ace
SEALKILNNIR
LB K38ace
LEVVVKER
LB K57ace
KLQQYQ
LB K83ace
EMLIADGIDPNELLQTMNATKVAGK
LB K87ace
VAGKTK
LB K107ace
YQYVDENGETKTWTGQGR
LB K120ace
TPAVIKNAIEEQGK
DMEM K6ace
SEALKILNNIR
DMEM K38ace
LEVVVKER
DMEM K83ace
EMLIADGIDPNELLQTMNATK
DMEM K107ace
YQYVDENGETKTWTGQGR
DMEM K120ace
TPAVIKNAIEEQGK
Relative Intensity (%)
M/Z

Figure S2

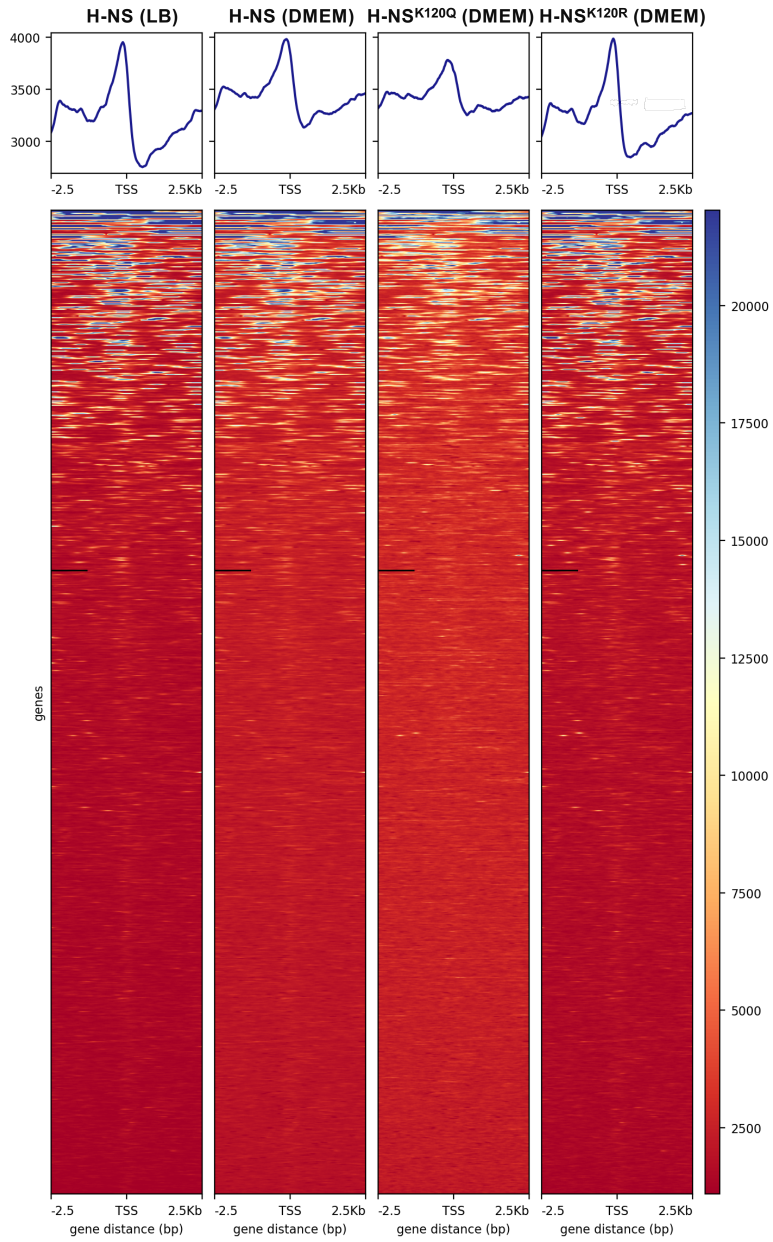
H-NS (LB)
H-NS (DMEM)
H-NS^{K120Q} (DMEM)
H-NS^{K120R} (DMEM)
4000
3500
3000
-2.5
TSS
2.5Kb
genes
gene distance (bp)
20000
17500
15000
12500
10000
7500
5000
2500

Figure S3

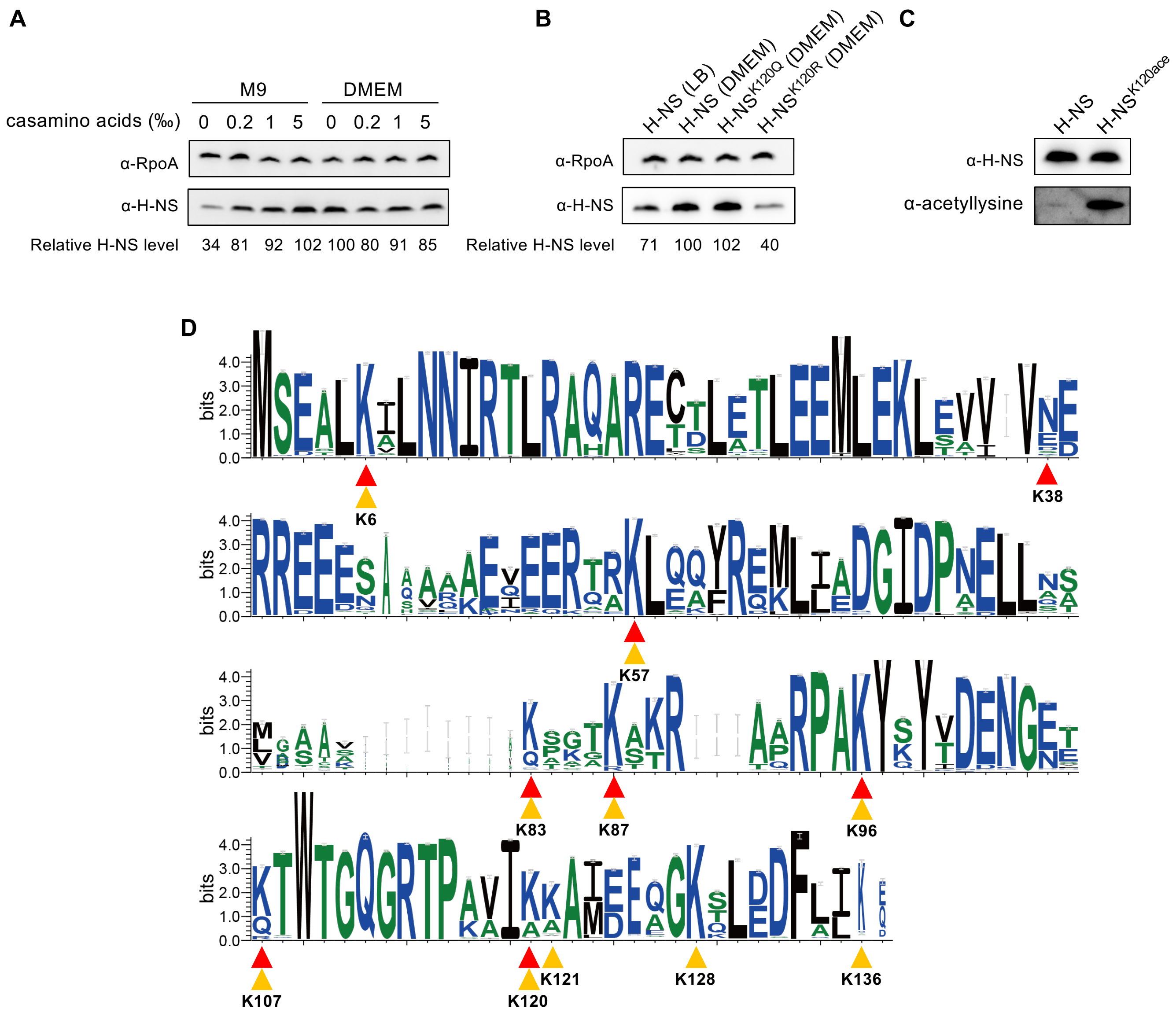
A
M9
DMEM
casamino acids (‰) 0 0.2 1 5 0 0.2 1 5
α-RpoA
α-H-NS
Relative H-NS level 34 81 92 102 100 80 91 85
B
H-NS (LB)
H-NS (DMEM)
H-NS K120Q (DMEM)
H-NS K120R (DMEM)
α-RpoA
α-H-NS
Relative H-NS level 71 100 102 40
C
H-NS
H-NS K120ace
α-H-NS
α-acetyllysine
D
bits
K6
K38
K57
K83
K87
K96
K107
K120
K121
K128
K136

Figure S4

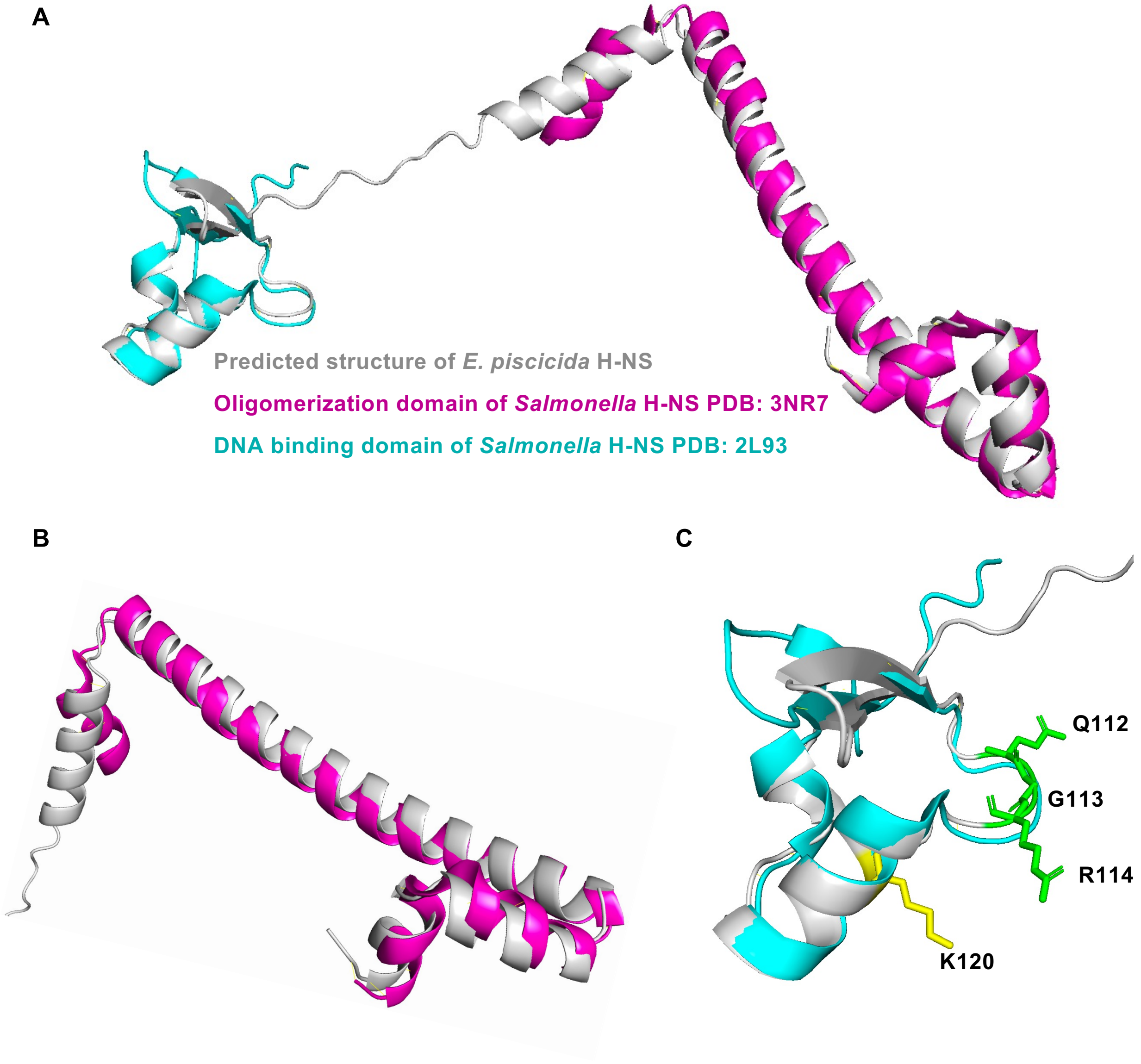
A
Predicted structure of E. piscicida H-NS
Oligomerization domain of Salmonella H-NS PDB: 3NR7
DNA binding domain of Salmonella H-NS PDB: 2L93
B
C
Q112
G113
R114
K120

Figure S5

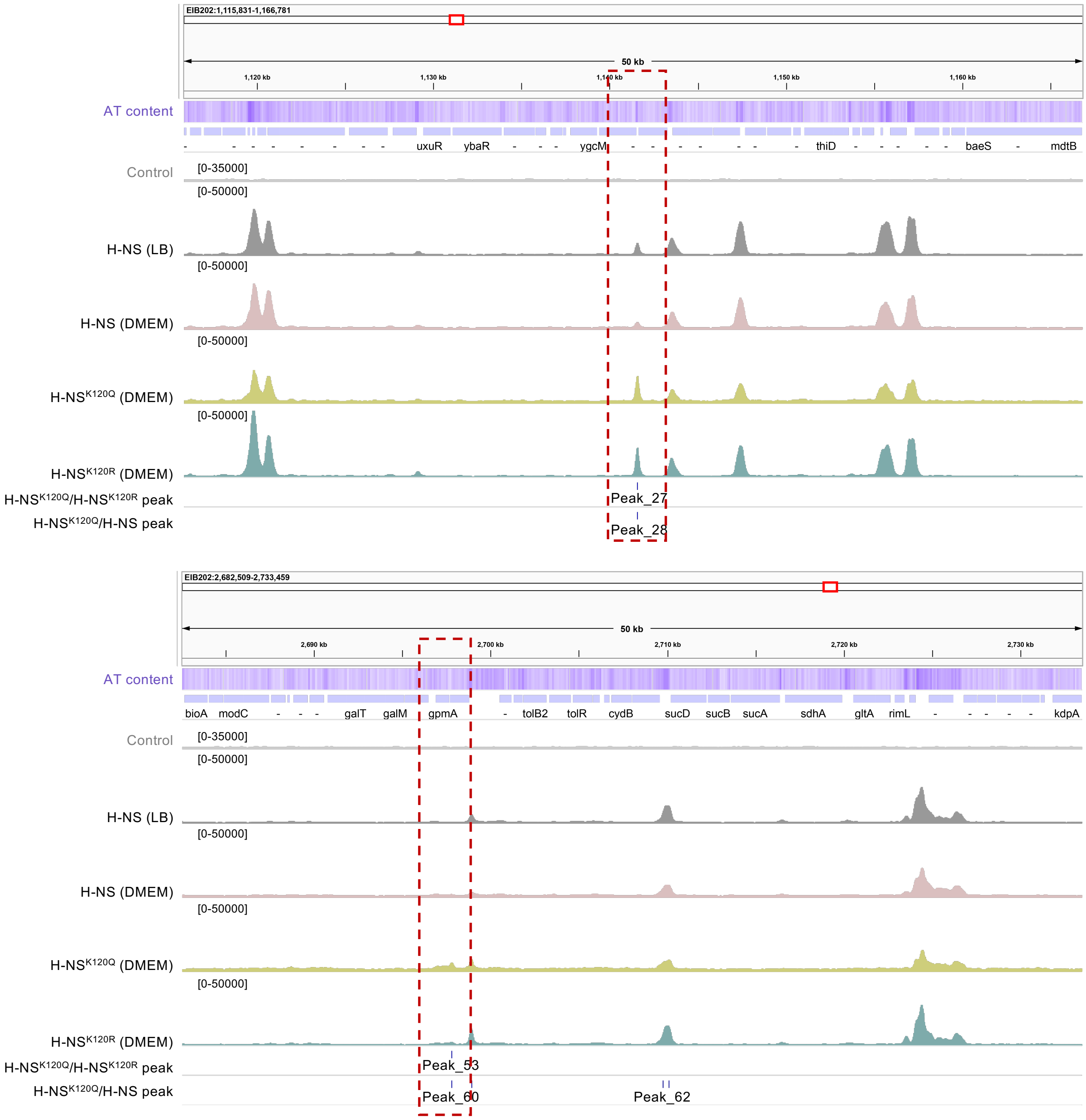
EIB202:1,115,831-1,166,781
50 kb
1,120 kb
1,130 kb
1,140 kb
1,150 kb
1,160 kb
AT content
uxuR
ybaR
ygcM
thiD
baeS
mdtB
Control
[0-35000]
[0-50000]
H-NS (LB)
H-NS (DMEM)
H-NS^{K120Q} (DMEM)
H-NS^{K120R} (DMEM)
H-NS^{K120Q}/H-NS^{K120R} peak
H-NS^{K120Q}/H-NS peak
Peak_27
Peak_28
EIB202:2,682,509-2,733,459
2,690 kb
2,700 kb
2,710 kb
2,720 kb
2,730 kb
bioA
modC
galT
galM
gpmA
tolB2
tolR
cydB
sucD
sucB
sucA
sdhA
gltA
rimL
kdpA
Peak_53
Peak_60
Peak_62

Figure S6
